# Age-Related Changes to Multisensory Integration and Audiovisual Speech Perception

**DOI:** 10.3390/brainsci13081126

**Published:** 2023-07-25

**Authors:** Jessica L. Pepper, Helen E. Nuttall

**Affiliations:** Department of Psychology, Lancaster University, Bailrigg LA1 4YF, UK; j.l.pepper@lancaster.ac.uk

**Keywords:** multisensory, ageing, speech, temporal binding, attention, inhibition, alpha activity, falls

## Abstract

Multisensory integration is essential for the quick and accurate perception of our environment, particularly in everyday tasks like speech perception. Research has highlighted the importance of investigating bottom-up and top-down contributions to multisensory integration and how these change as a function of ageing. Specifically, perceptual factors like the temporal binding window and cognitive factors like attention and inhibition appear to be fundamental in the integration of visual and auditory information—integration that may become less efficient as we age. These factors have been linked to brain areas like the superior temporal sulcus, with neural oscillations in the alpha-band frequency also being implicated in multisensory processing. Age-related changes in multisensory integration may have significant consequences for the well-being of our increasingly ageing population, affecting their ability to communicate with others and safely move through their environment; it is crucial that the evidence surrounding this subject continues to be carefully investigated. This review will discuss research into age-related changes in the perceptual and cognitive mechanisms of multisensory integration and the impact that these changes have on speech perception and fall risk. The role of oscillatory alpha activity is of particular interest, as it may be key in the modulation of multisensory integration.

## 1. Multisensory Integration

Multisensory integration encompasses the mechanisms involved when information is processed by individual sensory systems and combined into a coherent perceptual event [1]. Accurate and reliable multisensory integration is dependent on the spatial and temporal proximity of stimuli from different modalities [2,3]. If visual and auditory inputs are presented closely together in time and space, there is an increased likelihood that they will be perceived as occurring from the same event and integrated, to the benefit of perceptual performance [4,5,6]. The precise and timely binding of congruent sensory information is therefore essential for enabling humans to make sense of their physical environment and successfully perform important everyday tasks [7,8,9], such as participating in hobbies, mobility and balance, and speech perception [10,11].

Not only do older adults experience declines in vision and hearing function, but age-related changes in neurobiology also result in differences in how people process sensory information; these changes have a significant impact on both our ability to perceive speech and our ability to safely move through our environment. Difficulties in understanding speech in noisy environments is a predominant complaint of older adults, which can negatively affect mental well-being due to withdrawing from social situations where speech perception is challenging [12,13]. This reduced socialisation is exacerbated by the difficulties in mobility associated with ageing; older adults experience an increased risk of falls compared to younger adults, which is intrinsically linked to age-related changes in multisensory processing. Over one-third of people over 65 will experience a fall this year, and on average, injuries caused by falls cost the UK’s National Health Service over GBP 4.4 billion each year [14]. Due to our increasingly ageing population, studying how the bottom-up and top-down mechanisms of multisensory integration change as a function of healthy ageing has become a high priority in current cognitive neuroscience research [7,15], with the aim of understanding how to potentially strengthen the efficacy of older adults’ perception and improve their ability to quickly and accurately interpret their dynamic, multisensory environment.

If visual and auditory inputs are congruent and should conceptually be bound together, the integration of these unisensory cues produces behavioural enhancements. Within both laboratory and naturalistic tasks, such enhancements often manifest as increased accuracy or faster reaction times in response to multisensory stimuli compared to unisensory stimuli [16,17,18]. The most significant multisensory benefits are often reported when the unisensory stimulus elicits a particularly weak or noisy response when presented on its own [19]. In other words, many researchers have concluded that multisensory enhancements are most apparent when the unisensory stimuli are ambiguous [16].

Previous research suggests that older adults display increased multisensory integration and greater multisensory enhancements relative to younger adults [16,20,21]. When presented with multisensory stimuli versus unisensory stimuli, older adults show greater increases in accuracy and speeded reaction times compared to baseline, more so than the enhancements exhibited by younger adults. It would be reasonable to initially suggest that the multisensory benefits that older adults exhibit could be due to the deterioration of sensory function that is associated with healthy ageing [18]. For example, as we get older, humans experience declines in visual acuity [22,23], which could manifest as older adults requiring more light to see clearly, having difficulty reading or focussing on nearby objects, and changes in colour perception [24]. In addition, older adults experience declines in auditory sensitivity at all frequencies, which results in a less accurate acoustic performance in everyday tasks such as speech discrimination [22,25,26]. Taken together, age-related declines in sensory function may mean that unisensory information may be noisy and must be supported by stimuli from a different modality [15,27,28]. As such, preliminary research in this area focussed on explaining older adults’ multisensory benefits through theories akin to the principle of inverse effectiveness—if the auditory or visual inputs are ambiguous due to age-related declines in vision and hearing, perhaps the gains produced when more sensory information is presented together are greater than when strong stimuli are presented individually [29]. However, after comparing participants’ reaction times in unisensory and multisensory discrimination trials to those predicted by the independent race model [30,31], Laurienti et al. [16] found that older adults’ multisensory enhancements could not be explained by age-related sensory impairments alone.

As such, whilst the exact reason as to how and why older adults exhibit such multisensory enhancements remains unknown, research is beginning to move away from the principle of inverse effectiveness as the sole explanation [16,25], creating space for emerging theories that provide a more comprehensive account of how healthy ageing can affect perception and action.

The objective of this narrative review is to examine how audiovisual integration for speech processing is affected by healthy ageing. Through critically analysing paradigms used in previous research and drawing together key findings, the following sections will provide a novel perspective on the associations between audiovisual integration, speech perception, and fall risk in older adults. To our knowledge, this is the first narrative review to explicitly highlight the shared attentional and perceptual mechanisms involved in speech perception and fall risk, with a predominant focus on the role of oscillatory alpha activity in audiovisual integration. Comprehensively combining neuroscientific research surrounding age-related changes in speech perception and fall risk should uncover important common factors regarding the modulation of audiovisual processing, in key real-world contexts. As such, this review will not only aid in providing directions for future research but will also underline the impact that multisensory changes jointly have on speech perception, mobility and the overall quality of life of older adults.

## 2. Temporal Precision in Multisensory Integration

A key bottom-up factor that influences whether two sensory inputs are bound together is their temporal proximity. The time range within which visual and auditory information can be presented, perceived as simultaneous and subsequently bound together is known as the temporal binding window (TBW). The TBW is a mechanism used by the brain to accept naturally occurring stimulus asynchrony (e.g., due to differences in the speeds of light and sound [32]); this means that if two sensory inputs should veridically be integrated due to occurring from the same event, they are able to be integrated, even if they are not processed at exactly the same time [5,33,34,35]. Crucially, as stimulus onset asynchrony (SOA) increases—the time difference between the presentation of visual stimuli and auditory stimuli—the likelihood of multisensory integration decreases [36,37]. This highlights the importance of the TBW in accurately and timely multisensory integration and the global perception of our environment. For example, with regards to speech perception, we produce the most accurate percept of the words being spoken to us when we combine the visual input of the speaker’s mouth moving with the auditory input of the words being vocalised—we can successfully bind these inputs together because they are congruent, they are spatially proximal, and they fall within the TBW [2,38].

An important finding in recent years is that older adults have a wider TBW than younger adults [33,39,40,41,42,43]. As such, the TBW could be fundamental in explaining why older adults demonstrate increased integration [44]. Ultimately, due to their wider TBW, older adults have a larger time frame over which integration can occur, thus displaying an increased likelihood of binding sensory signals that are asynchronous and failing to ignore incongruent information [5]. Likewise, due to the narrower TBW of younger adults, they demonstrate greater temporal precision in tasks where successful performance depends upon segregating asynchronous audiovisual inputs—that is, identifying when stimuli from two different modalities should remain separate [5].

## 3. How Do We Measure Audiovisual Integration?

Mechanisms that impact multisensory integration, like the TBW, are often studied in research through the use of psychophysical illusions. For example, in the sound-induced flash illusion, participants are presented with a single visual flash and two auditory beeps, and are asked to report the number of flashes they observed [45]; when the visual and auditory inputs are presented in close temporal proximity, the multisensory illusion induces the perception that two flashes are presented rather than one [45]. Setti et al. [46] and Hirst et al. [28] implemented the sound-induced flash illusion and found that older adults were more susceptible to the illusory effects at longer SOAs than younger adults, integrating visual and auditory information more frequently than younger adults even though the inputs were not temporally aligned [5]. The illusion indicates the maximum SOAs in which stimuli can be presented and still be integrated [43], as well as highlighting the difficulties older adults have in discriminating temporal order and simultaneity compared to younger adults [42,46]. Some researchers have postulated that the increased susceptibility to the sound-induced flash illusion in older adults may be due to an increased reliance on multisensory integration, compensating for weak unisensory information due to age-related sensory declines, which is in line with theories like the principle of inverse effectiveness [7,47,48,49]. However, recent criticisms from Basharat et al. [50] suggest that the sound-induced flash illusion may not be a sufficiently sensitive measure, potentially underestimating the extent to which multisensory integration can occur.

An alternative psychophysical illusion that appears to be generating increasing support for its ability to provide insight into the bottom-up and top-down mechanisms involved in multisensory integration is the stream–bounce illusion, which uses dynamic rather than static stimuli and thus may provide a more ecologically valid indication of how people may perceive their dynamic everyday environment [51,52]. In the illusion, if an irrelevant sound is played at the same time as two moving circles touch, participants are more prone to binding the visual intersection and the auditory tone together, resulting in the percept of the circles bouncing off each other. Increasing the SOA between the sound playing and the circles touching generally decreases the likelihood of participants perceiving the circles to bounce [53]. This example of audiovisual integration is a phenomenon known as the auditory bounce effect [53,54,55,56]. Importantly, brain regions believed to be involved in multisensory integration, such as the superior colliculus and the posterior parietal cortex, display increased activation when the circles are perceived to bounce compared to when they are perceived to stream [55], as well as transcranial magnetic stimulation (TMS) to the right posterior parietal cortex decreasing the likelihood of the participant perceiving the circles to bounce [56]. As such, the stream–bounce illusion has proven to be a highly useful paradigm to investigate both the perceptual and cognitive elements of dynamic multisensory integration.

Focussing specifically on speech perception, arguably the most renowned illusion used to measure audiovisual integration is the McGurk effect [57]. In the McGurk effect, simultaneously presenting the visual input of a speaker articulating the sound/ga/with the auditory input of/ba/often results in the fused “McGurk” percept of “da” [58], indicating that participants bound the incongruent visual and auditory inputs together. Measuring susceptibility to the McGurk effect in different populations, and in clear or noisy listening environments, allows researchers to draw comparisons regarding the extent of multisensory integration between groups. Older adults may exhibit an enhanced McGurk effect compared to younger adults—when auditory and visual inputs are incongruent (and therefore should, in theory, remain separate), older adults bind these inputs together more frequently than younger adults do [59,60]. More than 71% of adults aged over 70 experience age-related hearing loss [12]; some researchers have hypothesised that to compensate for this, older adults may allocate more attentional resources to alternative modalities, like vision, to interpret acoustic information [59,61,62,63]. Indeed, older adults may undergo cross-modal cortical re-organisation due to age-related hearing loss, whereby auditory cortical regions such as the superior temporal gyrus receive reduced stimulation and may be more extensively recruited by the visual modality [12,64,65,66,67]. The increased resources available to the visual modality means that older adults may be able to rely on vision to support the auditory system in disambiguating speech [12,64,67,68,69,70]. In the McGurk effect, increased attention to visual inputs would result in a higher number of fused McGurk percepts in older adults.

However, at this point, it is important to note that many studies have found a similar susceptibility to the McGurk effect between younger and older adults. Some researchers have noted that the unisensory declines that naturally occur with healthy ageing, and the individual differences in factors such as education levels of participants, mean that comparisons between younger and older adults regarding their multisensory integration in a McGurk task can be challenging due to variability within age groups [15,44,59,71,72,73]. In addition, the McGurk has been criticised as being too simplistic and abstract in its representation of how multisensory speech perception happens in everyday life [74,75,76]. For example, the use of individual syllables and incongruent auditory and visual inputs are not elements that listeners experience in naturalistic conversations, casting doubt on whether the McGurk effect is an ecologically valid way to study veridical speech perception [74,75,77].

As such, some researchers are beginning to move away from McGurk as a measure of audiovisual speech perception and have instead explored alternative ways in which realistic multisensory integration can be investigated (see [74] for a review). For example, Peelle et al. [78] conducted an fMRI study in which auditory-only, visual-only and audiovisual whole words were presented in differing levels of background noise; the researchers found that the functional connectivity between the visual cortex and the auditory cortex was stronger in audiovisual conditions than in the unisensory conditions, a neural indication that participants were binding visual and auditory inputs together. Applying background noise increases the ambiguity of auditory information and is therefore an effective way to engage and subsequently measure multisensory integration in challenging conditions due to the increased reliance on vision [74,78]. Indeed, this technique can also be applied to visual paradigms, manipulating the clarity of the visual input (e.g., increasing blurriness) and measuring the effect on multisensory integration due to the increased reliance on audition [74,79]. Whilst age-related changes in speech-in-noise perception have regularly been investigated using auditory-only paradigms, there is also a large amount of important research analysing how visual information is used to support the auditory system in disambiguating acoustic information in noisy environments. Given the multisensory focus of the current review, we predominantly examine speech perception experiments that have implemented audiovisual paradigms.

As discussed earlier (p. 2), combining congruent visual and auditory information results in multisensory enhancements—audiovisual information has been found to improve the ability to perceive speech compared to unisensory inputs or incongruent audiovisual inputs [73,80,81]. However, research into age-related changes in audiovisual integration for speech perception has generated mixed findings, particularly due to individual differences in unisensory acuity and cognitive function between participants, and the type of speech stimuli used (e.g., full words, full sentences or phonemes [82,83]). Certainly, for simple stimuli such as flashes and beeps, older adults are able to compensate for age-related declines in visual acuity and hearing sensitivity by integrating information from each modality to produce a quick and accurate multisensory performance [16,82]. However, in more complex scenarios like speech perception in ambiguous conditions, age-related declines in audiovisual integration become apparent [80,84]. For example, Tye-Murray et al. [85] and Gordon and Allen [86] found that whilst younger and older adults displayed equivalent multisensory enhancements when presented with clear audiovisual speech stimuli, older adults showed smaller multisensory enhancements compared to younger adults when the congruent visual inputs were degraded. Similarly, in an audiovisual speech-in-noise task, Stevenson et al. [82] found that older adults showed smaller multisensory benefits compared to younger adults for whole-word recognition when the auditory inputs were degraded (i.e., signal-to-noise ratio was lower); however, for the easier task of phoneme recognition, older and younger adults displayed equivalent increases in multisensory benefits in noisy listening environments. Not only do these findings highlight how audiovisual integration can serve as a compensatory mechanism used to facilitate speech perception, but they also indicate that the ability to detect age-related changes in audiovisual speech perception is dependent upon the complexity of speech stimuli implemented in each experimental paradigm. Future researchers must be mindful of designing a speech perception task that is too simplistic and consider taking steps to avoid ‘ceiling effects’; easy speech perception tasks with a very high accuracy rate make it difficult to identify significant differences in performance between unisensory and audiovisual conditions, and differences between younger and older adults in their multisensory enhancements [80,87,88].

Each of the psychophysical methods discussed have their critics; however, they have provided valuable contributions in measuring the extent to which visual and auditory information can be integrated and the temporal factors that influence such integration. It is likely that utilising paradigms that involve dynamic stimuli (like the stream–bounce illusion), or that reflect naturalistic speech perception, would produce results with more real-world resemblance than experiments that simply use static flashes and beeps; this would provide greater insight into how the perceptual changes that come with healthy ageing affect the ability of older adults to successfully navigate through their dynamic, multisensory environment [89].

## 4. Attentional Modulation of Audiovisual Integration

If older adults are prone to erroneous increased integration, it is important to study whether there are any mechanisms or processes that could be employed to modulate the multisensory integration of older adults, improving their precision by reducing the influence of irrelevant sensory information. One potential top-down mechanism that is generating increasing interest with regards to the modulation of multisensory integration is attentional control [90]. Specifically, selective attention is believed to enhance the perception of sensory information that is task-relevant and suppress the processing of noisy, irrelevant sensory information that should not be incorporated into the percept [91,92]. When multiple sensory modalities are receiving lots of competing inputs, top-down selective attention is essential for multisensory integration between the congruent stimuli [1,93]. If multimodal inputs are congruent, multisensory integration is facilitated (i.e., more accurate responses, faster reaction times); however, if the inputs are incongruent, attention can correctly impede integration [1]. For example, in a multisensory fMRI study involving audiovisual speech, Fairhall and Macaluso [94] found that when attention was directed towards visual lip movements that were congruent with the auditory sentence being played, this improved performance and resulted in increased activation in multisensory brain areas such as the superior temporal sulcus and the superior colliculus, compared to the brain activity when attention was directed towards incongruent lip-movements.

These attentional mechanisms are clearly highly relevant to multisensory speech perception and how our ability to integrate audiovisual information may change as a function of ageing [90,95]—attention to relevant inputs and inhibition of irrelevant inputs are crucial in the quick and accurate processing of audiovisual speech [87,96,97]. For example, it is well-established that under cocktail-party conditions (i.e., segregating and attending to one speech source amongst multiple speakers [98]), accurate speech perception requires the listener to simultaneously direct attentional resources to the target speaker and suppress the distracting, irrelevant information of background speakers or other external noise in the environment [99,100].

The ability to inhibit distracting and irrelevant information in situations like this may weaken as we grow older [101,102,103,104,105,106,107,108]. This is known as the inhibitory deficit hypothesis—an increased processing and subsequent integration of irrelevant sensory information due to the reduced ability to ignore [109]. If this is the case for older adults, perhaps the weakening of inhibitory abilities could be an explanatory factor for their reduced speech-in-noise perception abilities; older adults may find it more difficult to ignore task-irrelevant information and therefore display increased integration relative to younger adults [87,110,111].

However, there is conflicting evidence for the theory that inhibition changes with healthy ageing—research conducted by Hugenschmidt et al. [112] and Guerreiro et al. [113,114,115] indicates that the ability to ignore distracting information is preserved with healthy ageing. As such, further research is required to determine whether older adults display weaker inhibitory abilities compared to younger adults when presented with visual and auditory information. At the moment, the mixed literature indicates that such findings may be both task-specific and modality-dependent. Nevertheless, if older adults potentially have difficulty in effectively deploying attentional resources to the task at hand, the subsequent reduced regulation of multisensory interactions could be the underlying cause of the wider (less restricted) TBW and the increased multisensory integration exhibited by older adults relative to younger adults [25].

## 5. Multisensory Integration, Attentional Control, and Falls

An emerging theory as to why older adults display increased multisensory integration is focussed on this combination of the potentially weaker inhibitory control and wider TBW of older adults and whether it is associated with their increased risk of falls [33,42,46]. As discussed, older adults may be inefficient in allocating the attentional resources required to sufficiently narrow the TBW and modulate sensory processing, resulting in increased integration of visual and auditory stimuli that may be asynchronous and irrelevant [44,46]. This can lead to dangerously inaccurate perceptions of an older adult’s environment, resulting in a fall.

It is important to note that there are many multifaceted risk factors associated with falls in older adults. For example, age-related muscle loss [116], medications that cause light-headedness or vertigo [117], and age-related unisensory declines [118,119] all contribute significantly to the weaker balance maintenance and increased risk of falls in older adults compared to younger adults. However, crucially, stable balance, alongside musculoskeletal demands, is also dependent upon the accurate integration of reliable sensory information in the brain [89,120]. In older adults, not only is balance made more challenging by declines in unisensory acuity, but declines in the efficacy and reliability of multisensory integration mechanisms like attentional control are also likely to negatively impact balance and fall risk. When fall-prone older adults are required to simultaneously maintain their balance and perform a multisensory task, such as focussing on perceiving speech whilst walking, the activity in the brain regions associated with balance (e.g., the somatosensory cortex [121,122]) may be reduced, whilst the brain regions associated with audiovisual speech perception (e.g., superior temporal sulcus [94,123]) may be more active [25,91,120]. The requirement to process speech diverts the attentional resources required for stable balance and results in a fall, a concept which is in line with the information degradation hypothesis. The hypothesis suggests that humans possess a limited amount of attentional resources; age-related degradations of auditory inputs place demand on these resources (i.e., there is an increased effort to process auditory information). This results in the diversion of cognitive resources, away from balance and towards tasks like speech-in-noise perception, for example, which require effortful listening [12].

The role of attention in balance maintenance and fall risk has therefore regularly been investigated using dual-task methodologies, assessing the impact that manipulating the attentional demands required for different postural conditions has on perceptual performance. For example, Lajoie et al. [124] asked participants to provide a verbal response to an auditory stimulus whilst sitting, standing, and walking and found that attentional costs were greater (i.e., perceptual performance was worse) in the walking condition compared to the seated and standing conditions. The authors concluded that more challenging balance conditions required a greater allocation of attentional resources, suggesting that balance maintenance loads the cognitive system and the attentional control required may detract from integrative processes required for other tasks, such as speech perception [124].

This is supported by research by Stapleton et al. [125], who asked participants to complete the sound-induced flash illusion whilst sitting and standing; they found that fall-prone older adults were more susceptible to the illusory effects when standing than when sitting, whilst healthy older adults did not show a difference in susceptibility to the illusion across the postural conditions. Stapleton et al. [125] argued that fall-prone older adults require more attentional resources to maintain balance, leaving fewer attentional resources for the multisensory sound-induced flash task and therefore displaying increased—less modulated—integration of visual and auditory inputs that were not temporally aligned (i.e., producing more illusory percepts). As such, dual-task multisensory integration in older adults may be dependent upon how they allocate the necessary attentional resources—to the multisensory task at hand, or to maintaining balance.

In sum, the cognitive mechanisms required for audiovisual integration are strongly associated with balance maintenance and fall risk. This highlights the importance of investigating how such mechanisms are affected by healthy ageing to understand the impact of age-related changes in multisensory integration. In order to gain a truly comprehensive understanding, it is necessary to study how the neurobiological basis of multisensory integration changes as a function of ageing—relying on behavioural data alone is not likely to provide a thorough explanation as to how the central nervous system processes and combines multisensory information.

## 6. Neurobiology of Multisensory Integration

Previous research has uncovered multiple sub-cortical and cortical brain regions associated with multisensory integration, which work together to bind visual and auditory information that is temporally, spatially, and semantically congruent (see [126,127] for recent meta-analyses identifying the implicated brain areas). The following sections of this review will focus on arguably the most renowned cortical region for audiovisual integration—the superior temporal sulcus (STS; [128])—as well as discussing the neural oscillations within sensory cortices that may play a key role in the modulation of multisensory processing.

From a neurobiological perspective, the STS is a clear target for research into audiovsiual integration due to its location at the junction between occipital and temporal cortex [32]. Neurons within the STS display strong activation during the processing of meaningful everyday visual and auditory stimuli, such as moving people or objects, or speech and language comprehension [78,128,129,130,131,132]. Activity in the STS also reflects the “super-additivity” theory in multisensory integration, in that bimodal stimuli elicit a greater neural response in this brain area than when unisensory stimuli are presented [128] (however, see [133]). Indeed, Wright et al. [132] found that whilst the posterior STS responded strongly to visual stimuli, and the anterior STS responded strongly to auditory stimuli, combined audiovisual stimuli provoked the strongest neural response throughout the STS. Crucially, brain imaging research has indicated that multisensory effects in the STS are greatest (i.e., neural activity is increased) when participants listen to speech in noise; participants use congruent visual information to support the noisy, ambiguous auditory information and subsequently facilitate speech perception [78,130,134,135,136,137].

The STS has also been implicated in specific mechanisms like the temporal binding window—researchers have presented participants with multisensory stimuli and analysed how temporal and spatial factors evoke activations in different brain areas [138,139,140]. For example, Calvert et al. [138] exposed participants to audiovisual stimuli in the form of a person reading a story, in which the visual and auditory inputs were either congruent or incongruent. The researchers found that the congruent condition, in which multisensory integration is quick and accurate due to the visual and auditory stimuli being temporally and semantically aligned, evoked a greater neural response in the STS compared to the incongruent condition. This is supported by research by Powers et al. [141], who not only found that perceptual training can narrow the TBW and improve the temporal precision of multisensory integration (as did [33]), but using fMRI, the researchers also found that these changes were reflected in increased activity in the STS. The STS is therefore strongly implicated in the evaluation of the temporal factors necessary for multisensory integration.

A growing body of evidence suggests that the STS plays a key role in the susceptibility to the McGurk effect, displaying increased bilateral activation when the incongruent auditory and visual inputs are bound together in the task [142]. This is supported by a fMRI-TMS study conducted by Beauchamp et al. [143], who found that perturbing neural activity in the STS reduced the number of McGurk responses provided by participants, and this perturbation did not influence responses to non-McGurk stimuli [63,143]. Overall, the STS is clearly implicated in the binding of visual and auditory information in the dynamic, multisensory environments that people must make sense of in everyday life scenarios like speech perception [32,133,140,144,145].

However, it is important to note that when comparing neural activity in younger and older adults during a McGurk task, fMRI data from Diaz and Yalcinbas [63] revealed that each age group engaged different brain regions and thus different mechanisms throughout the task. Younger adults relied heavily on sensory cortices such as the superior temporal gyrus, whereas older adults were more likely to utilise frontal brain regions including the superior frontal gyrus (involved in executive function) and the superior parietal lobule (involved in attentional control). This indicates that older adults required more cognitive resources than younger adults to perceive audiovisual speech, once again highlighting the important role of cognitive control in multisensory processing [90]. The authors suggested, in line with previous research discussed in this review, that older adults may rely on alternative strategies to perceive audiovisual speech as a potential compensatory mechanism for declines in sensory function [7,28,48,63]. Fundamentally, these data suggest that the STS is not exclusive for its role in multisensory integration, and it is instead highly likely that multiple different sensory and cognitive brain areas are functionally connected (e.g., the superior parietal cortex, prefrontal cortex, premotor cortex; [78,127,146]), working together to bind visual and auditory inputs in younger and older adults for quick and accurate performance in tasks like speech perception and balance maintenance.

## 7. Oscillatory Alpha Activity in Multisensory Integration

Whilst the specific brain regions associated with bottom-up and top-down multisensory integration have been well-established using techniques such as fMRI and PET, arguably less is known about the neural oscillations involved in multisensory integration and how these may change with healthy ageing. Neural oscillations are a compelling area of research due to their ability to index the synchronisation of brain activity within and across cortical areas, providing crucial insight into the neurophysiology of perception and cognition [147]. Due to the fact that multisensory integration engages multiple different brain regions, oscillatory activity can reveal how these areas coordinate with each other to facilitate perception. Specifically, oscillations reflect neural activity on a population level [148], providing a direct indication of the brain areas that simultaneously exhibit increased activation during the processing and binding of audiovisual information. Using neural oscillations, it is therefore possible to examine, with high temporal accuracy, the activity and functional connectivity in different brain regions during multisensory integration [149] and how this may change with healthy ageing. In addition, in the same way that we can link different brain regions like the STS to certain functions, oscillations in different frequency bands (alpha, beta, gamma, and theta) are believed to be responsible for specific mechanisms; bottom-up sensory processing is often associated with gamma-band activity (greater than 30 Hz), whereas top-down modulation of sensory processing is linked to lower frequency bands (less than 30 Hz) [149,150]. Studying simultaneous fluctuations in oscillatory activity within these frequency bands during behavioural tasks allows conclusions to be drawn regarding their roles in perception, cognition, and action [149]. An area of research which is generating increasing interest is the link between attentional control, multisensory integration, and cortical oscillations in the alpha band (8–12 Hz). Historically, alpha oscillations have often been referred to as “idling” rhythms, indicative of resting brain areas. However, oscillatory alpha activity is also strongly associated with the top-down processes involved in multisensory integration such as selective attention [151,152].

Increases in alpha activity, particularly in parieto-occipital regions, are believed to reflect the effort required to suppress distracting, task-irrelevant sensory information [151,152,153,154,155,156,157]; likewise, decreases in alpha power are indicative of increased neural activation in sensory brain regions, facilitating sensory processing [152,158,159]. When participants are directed towards an area of space in which the target stimulus is presented, alpha power decreases in parieto-occipital regions contralateral to the attended location [160,161] and increases in ipsilateral parieto-occipital brain regions [151,158,162,163]. In this way, attentional cuing tasks akin to those used by Posner et al. [164] have been implemented with unisensory and multisensory stimuli, comparing alpha power in the “attending” hemisphere to alpha power in the “ignoring” hemisphere during the task to analyse the participant’s ability to inhibit task-irrelevant information [111,152]. Taken together, this highlights alpha-band oscillations as a clear target for analysis of neural activity during multisensory processing under different attentional conditions.

Crucially, due to the hypothesised role of alpha in selective attention, and the deterioration of inhibitory abilities with healthy ageing, it is fair to suggest that younger and older adults may display age-related differences in alpha activity [102,103,107,108]. Borghini et al. [108] designed a transcranial alternating current stimulation (tACS) experiment to causally link age-related changes in alpha oscillations to inhibitory performance during a working memory task that required participants to ignore task-irrelevant information. Not only did the researchers confirm previous findings that inhibitory abilities were weaker in older adults, but also, Borghini et al. [108] found that stimulating alpha-band activity in the parietal region of older adults improved their inhibitory performance, to the extent that they were equally successful in the task as younger adults. These important findings indicate a clear link between alpha oscillations and inhibitory control; an age-related reduction in alpha activity in older adults may result in their weaker ability to ignore task-irrelevant information. Stimulation increased alpha activity and older adults subsequently displayed improvements in inhibitory control [108]. The findings of Borghini et al. [108] are a positive indication that whilst alpha activity and inhibitory abilities may diminish as we age, they could indeed be modulated through brain stimulation.

The role of oscillatory alpha power has also been studied in relation to speech perception in noisy environments. For example, O’Sullivan et al. [165] analysed participants’ alpha activity under cocktail-party conditions [98] and manipulated whether audiovisual inputs were congruent or incongruent; they found that alpha activity over the parieto-occipital brain regions could indicate whether the participant was attending to the visual modality or the auditory modality. That is, when successful performance in the task required participants to ignore incongruent visual information, EEG data displayed increases in alpha activity over parieto-occipital electrodes. In addition, in the condition where visual and auditory information was congruent, alpha activity decreased—both sensory modalities were receiving task-relevant information that facilitated speech processing, there was no distracting sensory input and therefore alpha activity was lower [152,165].

Recent research has therefore investigated whether the weaker performance of older adults in speech-in-noise tasks may be reflected in age-related differences in alpha power. For example, Tune et al. [166] asked middle-aged and older adults to complete a dichotic listening task in a noisy acoustic environment. Interestingly, the researchers found that on a neural level, middle-aged and older adults showed a similar modulation of alpha power, and on a behavioural level, both age groups performed similarly in the task [166]. Contrary to evidence suggesting that older adults may have inhibitory deficits, these findings suggest that selective spatial attention may be preserved with healthy ageing [166,167]. Tune et al. [166] also highlighted the high level of variability between participants when measuring data as sensitive as alpha power, finding that other cognitive characteristics of participants, such as education and working memory, were stronger predictors of behavioural performance than age. Indeed, Stern et al. [168] explained the importance of lifestyle and experiences in the ability to compensate for age-related declines in cognitive processes like attention. Namely, engaging in more social activities or education throughout our lives, for example, accumulates cognitive “reserve”, a resource bank which allows for the use of alternative cognitive strategies and which strengthens existing brain networks (see [68] for a detailed review). Individual differences in cognitive reserve would result in a mixed performance between younger and older adults in tasks that require inhibitory processes and attentional control, like speech-in-noise tasks. This once again highlights the importance of accounting for the sensory and cognitive individual differences of participants in multisensory research, especially when studying the sensitive age-related changes in such processes [28,44].

There is also evidence suggesting that the alpha band is strongly associated with temporal elements of multisensory processing, researched through the implementation of some of the illusions discussed earlier in this review. Crucially, Klimesch et al. [157] argued that alpha oscillations are responsible for the creation of time ranges in which sensory processing can occur, reflective of the concept of the TBW. This is supported by theories posited by Jensen and Mazaheri [169] and Ruhnau et al. [170]; oscillatory alpha may control the temporal processing of sensory information by establishing the temporal boundaries in which processing can occur after stimulus presentation. In other words, some researchers have suggested that the length of the oscillatory cycle directly relates to the TBW for multisensory integration, in which individual alpha frequency could predict the susceptibility to audiovisual illusory percepts. For example, Cecere et al. [171] hypothesised that the duration of an alpha oscillation could index the temporal window for the integration of visual and auditory information in the sound-induced flash illusion. The researchers found a positive correlation between individual alpha frequency (IAF) and the TBW at which the illusion could be maximally perceived; a lower IAF produced a longer TBW for multisensory integration to occur [155], increasing the susceptibility to the illusion at longer SOAs. This finding was replicated by Keil and Senkowski [155], who implemented the same paradigm and found that the length of the individual alpha band cycle in participants’ occipital cortex indexed the TBW for multisensory integration, further highlighting the important role that oscillatory alpha activity plays in audiovisual integration—both with respect to attentional control and temporal processing [151].

The critical finding, with respect to multisensory integration, is that oscillatory alpha activity appears to impact perception by modulating the excitability of the sensory cortices [151]. When cortical excitability is high (i.e., alpha power is low), neurons within that brain region are more likely to be activated resulting in increased multisensory integration.

## 8. Oscillatory Alpha Activity in Balance Maintenance and Fall Risk

As mentioned previously, balance maintenance and postural control are dependent upon the accurate integration of visual, auditory, proprioceptive and vestibular information [172]. Over recent years, it has been argued that cortical brain regions become increasingly involved in balance due to age-related declines in sub-cortical (cortico-thalamic) sensorimotor tracts and sensory deterioration [173,174,175]. As such, age-related changes in cortical frequency band activity are likely to uncover underlying neural reasons behind the increased risk of falls in older adults. In contrast to methods like fMRI, EEG can measure neural activity whilst participants are seated, standing, walking, or lying down, rendering it an incredibly useful technique to study how the different frequency bands contribute to balance maintenance by manipulating posture [175]. Whilst research has found that, under difficult balance conditions, there is increased activity in the theta band over parietal [176] and frontal [177] brain areas, alpha oscillations once again appear to be the cortical frequency band most highly associated with the multisensory, attentional aspects of balance.

For example, Edwards et al. [175] monitored alpha band activity whilst their sample of younger adult participants completed balance tasks of varying difficulty. As balance conditions became more challenging, the researchers found that alpha power decreased in central and parietal brain regions, reflecting the increased cortical excitability during balance maintenance [175]. The decreases in alpha power that Edwards et al. [175] found in the central and parietal brain regions during difficult balance tasks suggests that these regions were allocated increased attentional resources required for postural control, further supporting the role of alpha band activity in the attentional modulation of multisensory integration and simultaneous balance maintenance [175,178].

Paradigms have also been designed to measure differences in oscillatory alpha activity between younger adults, non-falling older adults, and older adults with a history of falls. Scurry et al. [179] implemented the sound-induced flash illusion with each of these groups; they measured oscillatory gamma activity (30–80 Hz) as an indicator of sensory processing and studied how this sensory processing is modulated by alpha activity, assessing the subsequent effect on susceptibility to the illusion. The researchers found that fall-prone older adults displayed a greater illusion strength than non-fall older adults and younger adults, which was a behavioural indication of increased multisensory integration in individuals who were at a greater risk of falls. Crucially, on a neural level, Scurry et al. [179] also found reduced phase-amplitude coupling between oscillatory alpha and gamma activity in fall-prone older adults compared to non-fall older adults and younger adults, which the researchers interpreted as a reduced top-down modulation of multisensory processing in fall-prone older adults. Taken together, it is likely that strong links exist between oscillatory alpha power and balance ability/fall risk, potentially due to the relationships both factors have with attentional control and multisensory integration. Studying more about these relationships and how they change as a function of ageing is key, with the aim of understanding how to improve the perception of and safe navigation through the dynamic everyday environment for older adults.

## 9. Concluding Remarks and Future Directions

This narrative review has highlighted current discussions emerging from research into the age-related changes in multisensory integration. Considering the fact that functions such as the temporal binding window and attentional control both appear to be susceptible to age-related declines, and both appear to have a significant influence on accurate and timely audiovisual integration, it is essential that they are not treated as mutually exclusive entities in terms of their influence on multisensory perception and how it changes across the lifespan [1,96].

The objective of this review was to provide a novel perspective on the shared mechanisms involved in audiovisual integration for speech processing and fall risk in older adults, as well as to investigate the role of oscillatory alpha activity in such mechanisms. The evidence reviewed suggests that speech perception becomes more difficult due to age-related changes in the modulation of audiovisual integration; weaker attentional control impacts older adults’ ability to suppress distractors and process only the most relevant, reliable sensory information when disambiguating speech. Likewise, these same attentional deficits that potentially exist in older adults appear to impede their balance; age-related changes in the ability to efficiently allocate attentional resources may be an underlying cognitive reason behind older adults’ increased risk of falls. Taken together, this review has highlighted how the top-down modulation of multisensory integration required to quickly, accurately, and safely interpret our environment may be significantly affected by healthy ageing, focussing on oscillatory alpha activity as the main neural correlate in attentional control, inhibition, and precise audiovisual integration. To our knowledge, this is the first review in which speech perception and fall risk have been considered in conjunction, to discuss the common cognitive and perceptual factors responsible for successful performance in each everyday task and how these change as a function of healthy ageing.

Using behavioural tasks such as the stream–bounce illusion or speech-in-noise paradigms, together with neuroscientific techniques like EEG, TMS, and fMRI, is a strong method for researchers to establish cause and effect associations between brain areas like the STS and the key processes required to bind auditory and visual inputs. As opposed to focussing on one single brain area in isolation, it is important that research shifts to acknowledge the numerous brain regions and frequency bands involved in multisensory integration, studying how they work together to perceive audiovisual events. The role of oscillatory alpha activity, in particular, appears to be a promising area of research due to its implication in the top-down modulation of multisensory processing; measuring neural oscillations like this allows for the investigation of how different brain areas coordinate to produce quick and accurate percepts of the environment. Analysing oscillatory activity across multiple cortical sites will provide crucial insights into how these areas are functionally connected and how this activity differs between younger and older adults. Throughout these studies, the individual differences of participants must be considered and minimised where possible, including variability in unisensory function, and in lifestyle factors like education and socialisation, which contribute to cognitive reserve. This would allow for accurate comparisons between age groups regarding how multisensory tasks like speech perception and balance maintenance are likely to develop as we age.

Given our increasingly ageing population, it is clear how important it is to research how multisensory integration changes with age and how this affects speech perception and incidence of falls, both of which have a significant impact on our quality of life [9,10]. A stronger understanding of age-related changes in multisensory integration may potentially lead to the development of cognitive treatments and therapies designed to strengthen the attentional control of older adults, improving their ability to quickly and accurately integrate relevant audiovisual information.

## Data Availability

There are no data associated with this article.

## References

[1] Talsma D., Senkowski D., Soto-Faraco S., Woldorff M.G. (2010). The multifaceted interplay between attention and multisensory integration. Trends Cogn. Sci..

[2] Vroomen J., Keetels M. (2010). Perception of intersensory synchrony: A tutorial review. Atten. Percept. Psychophys..

[3] Hillock A.R., Powers A.R., Wallace M.T. (2011). Binding of sights and sounds: Age-related changes in multisensory temporal processing. Neuropsychologia.

[4] Zampini M., Guest S., Shore D.I., Spence C. (2005). Audio-visual simultaneity judgments. Percept. Psychophys..

[5] Stevenson R.A., Zemtsov R.K., Wallace M.T. (2012). Individual differences in the multisensory temporal binding window predict susceptibility to audiovisual illusions. J. Exp. Psychol. Hum. Percept. Perform..

[6] Parise C.V., Harrar V., Ernst M.O., Spence C. (2013). Cross-correlation between Auditory and Visual Signals Promotes Multisensory Integration. Multisensory Res..

[7] de Dieuleveult A.L., Siemonsma P.C., van Erp J.B.F., Brouwer A.-M. (2017). Effects of Aging in Multisensory Integration: A Systematic Review. Front. Aging Neurosci..

[8] Higgen F.L., Heine C., Krawinkel L., Göschl F., Engel A.K., Hummel F.C., Xue G., Gerloff C. (2020). Crossmodal Congruency Enhances Performance of Healthy Older Adults in Visual-Tactile Pattern Matching. Front. Aging Neurosci..

[9] Cavazzana A., Röhrborn A., Garthus-Niegel S., Larsson M., Hummel T., Croy I. (2018). Sensory-specific impairment among older people. An investigation using both sensory thresholds and subjective measures across the five senses. PLoS ONE.

[10] Fischer M.E., Cruickshanks K.J., Klein B.E., Klein R., Schubert C.R., Wiley T.L. (2009). Multiple sensory impairment and quality of life. Ophthalmic Epidemiol..

[11] Bucks R.S., Ashworth D.L., Wilcock G.K., Siegfried K. (1996). Assessment of activities of daily living in dementia: Development of the Bristol Activities of Daily Living Scale. Age Ageing.

[12] Slade K., Plack C.J., Nuttall H.E. (2020). The Effects of Age-Related Hearing Loss on the Brain and Cognitive Function. Trends Neurosci..

[13] Weissgerber T., Müller C., Stöver T., Baumann U. (2022). Age differences in speech perception in noise and sound local-ization in individuals with subjective normal hearing. Front. Psychol..

[14] Office for Health Improvement and Disparities (2022, February). Falls: Applying All Our Health. Gov.uk. https://www.gov.uk/government/publications/falls-applying-all-our-health/falls-applying-all-our-health.

[15] Park H., Nannt J., Kayser C. (2021). Sensory- and memory-related drivers for altered ventriloquism effects and aftereffects in older adults. Cortex.

[16] Laurienti P.J., Burdette J.H., Maldjian J.A., Wallace M.T. (2006). Enhanced multisensory integration in older adults. Neurobiol. Aging.

[17] Laurienti P.J., Kraft R.A., Maldjian J.A., Burdette J.H., Wallace M.T. (2004). Semantic congruence is a critical factor in multisensory behavioral performance. Exp. Brain Res..

[18] Jones S.A., Beierholm U., Meijer D., Noppeney U. (2019). Older adults sacrifice response speed to preserve multisensory integration performance. Neurobiol. Aging.

[19] Meredith M.A., Stein B.E. (1983). Interactions Among Converging Sensory Inputs in the Superior Colliculus. Science.

[20] Peiffer A.M., Mozolic J.L., Hugenschmidt C.E., Laurienti P.J. (2007). Age-related multisensory enhancement in a simple audiovisual detection task. Neuroreport.

[21] Mahoney J.R., Li P.C.C., Oh-Park M., Verghese J., Holtzer R. (2011). Multisensory integration across the senses in young and old adults. Brain Res..

[22] Liu X.Z., Yan D. (2007). Ageing and hearing loss. J. Pathol. A J. Pathol. Soc. Great Br. Irel..

[23] Klein B.E., Moss S.E., Klein R., Lee K.E., Cruickshanks K.J. (2003). Associations of visual function with physical outcomes and limitations 5 years later in an older population: The Beaver Dam eye study. Ophthalmology.

[24] American Optometric Association (n.d). Adult Vision: 41 to 60 Years of Age. https://www.aoa.org/healthy-eyes/eye-health-for-life/adult-vision-41-to-60-years-of-age?sso=y.

[25] Mozolic J.L., Hugenschmidt C.E., Peiffer A.M., Laurienti P.J. (2012). Multisensory integration and aging. The Neural Bases of Multisensory Processes.

[26] Baraldi G.D.S., Almeida L.C.D., Borges A.C.D.C. (2007). Hearing loss in aging. Rev. Bras. Otorrinolaringol..

[27] Trelle A.N., Henson R.N., Simons J.S. (2019). Neural evidence for age-related differences in representational quality and strategic retrieval processes. Neurobiol. Aging.

[28] Hirst R.J., Setti A., Kenny R.A., Newell F.N. (2019). Age-related sensory decline mediates the Sound-Induced Flash Illusion: Evidence for reliability weighting models of multisensory perception. Sci. Rep..

[29] Meredith M.A., Stein B.E., Caruso V.C., Pages D.S., Sommer M.A., Groh J.M., Krüger H.M., Collins T., Englitz B., Cavanagh P. (1986). Visual, auditory, and somatosensory convergence on cells in superior colliculus results in multisensory integration. J. Neurophysiol..

[30] Miller J. (1982). Divided attention: Evidence for coactivation with redundant signals. Cogn. Psychol..

[31] Miller J. (2016). Statistical facilitation and the redundant signals effect: What are race and coactivation models?. Attention, Perception, Psychophys..

[32] Wallace M.T., Stevenson R.A. (2014). The construct of the multisensory temporal binding window and its dysregulation in developmental disabilities. Neuropsychologia.

[33] McGovern D.P., Burns S., Hirst R.J., Newell F.N. (2022). Perceptual training narrows the temporal binding window of audiovisual integration in both younger and older adults. Neuropsychologia.

[34] Mégevand P., Molholm S., Nayak A., Foxe J.J. (2013). Recalibration of the Multisensory Temporal Window of Integration Results from Changing Task Demands. PLoS ONE.

[35] Pöppel E., Schill K., von Steinbüchel N. (1990). Multistable states in intrahemispheric learning of a sensorimotor task. Neuroreport Int. J. Rapid Commun. Res. Neurosci..

[36] Vatakis A., Spence C. (2006). Audiovisual synchrony perception for music, speech, and object actions. Brain Res..

[37] Vatakis A., Spence C. (2007). Crossmodal binding: Evaluating the “unity assumption” using audiovisual speech stimuli. Percept. Psychophys..

[38] Van Wassenhove V., Grant K.W., Poeppel D. (2007). Temporal window of integration in auditory-visual speech perception. Neuropsychologia.

[39] Basharat A., Adams M.S., Staines W., Barnett-Cowan M. (2018). Simultaneity and Temporal Order Judgments Are Coded Differently and Change With Age: An Event-Related Potential Study. Front. Integr. Neurosci..

[40] Noel J.-P., De Niear M., Van der Burg E., Wallace M.T. (2016). Audiovisual Simultaneity Judgment and Rapid Recalibration throughout the Lifespan. PLoS ONE.

[41] Bedard G., Barnett-Cowan M. (2016). Impaired timing of audiovisual events in the elderly. Exp. Brain Res..

[42] Setti A., Stapleton J., Leahy D., Walsh C., Kenny R.A., Newell F.N. (2014). Improving the efficiency of multisensory integration in older adults: Audio-visual temporal discrimination training reduces susceptibility to the sound-induced flash illusion. Neuropsychologia.

[43] Diederich A., Colonius H., Schomburg A. (2008). Assessing age-related multisensory enhancement with the time-window-of-integration model. Neuropsychologia.

[44] Brooks C.J., Chan Y.M., Anderson A.J., McKendrick A.M. (2018). Audiovisual Temporal Perception in Aging: The Role of Multisensory Integration and Age-Related Sensory Loss. Front. Hum. Neurosci..

[45] Shams L., Ma W.J., Beierholm U. (2005). Sound-induced flash illusion as an optimal percept. Neuroreport.

[46] Setti A., Burke K.E., Kenny R.A., Newell F.N. (2011). Is inefficient multisensory processing associated with falls in older people?. Exp. Brain Res..

[47] DeLoss D.J., Pierce R.S., Andersen G.J. (2013). Multisensory integration, aging, and the sound-induced flash illusion. Psychol. Aging.

[48] de Boer-Schellekens L., Vroomen J. (2014). Multisensory integration compensates loss of sensitivity of visual temporal order in the elderly. Exp. Brain Res..

[49] Parker J.L., Robinson C.W. (2018). Changes in multisensory integration across the life span. Psychol. Aging.

[50] Basharat A., Mahoney J.R., Barnett-Cowan M. (2019). Temporal Metrics of Multisensory Processing Change in the Elderly. Multisensory Res..

[51] Matsuno T., Tomonaga M. (2011). Stream/bounce perception and the effect of depth cues in chimpanzees (*Pan troglodytes*). Atten. Percept. Psychophys..

[52] Sekuler R., Sekuler A.B., Lau R. (1997). Sound alters visual motion perception. Nature.

[53] Donohue S.E., Green J.J., Woldorff M.G. (2015). The effects of attention on the temporal integration of multisensory stimuli. Front. Integr. Neurosci..

[54] Watanabe K., Shimojo S. (2001). When Sound Affects Vision: Effects of Auditory Grouping on Visual Motion Perception. Psychol. Sci..

[55] Bushara K.O., Hanakawa T., Immisch I., Toma K., Kansaku K., Hallett M. (2003). Neural correlates of cross-modal binding. Nat. Neurosci..

[56] Maniglia M., Grassi M., Casco C., Campana G. (2012). The origin of the audiovisual bounce inducing effect: A TMS study. Neuropsychologia.

[57] Mcgurk H., Macdonald J. (1976). Hearing lips and seeing voices. Nature.

[58] Kraus N., Slater J. (2015). Music and language: Relations and disconnections. Handb. Clin. Neurol..

[59] Sekiyama K., Soshi T., Sakamoto S. (2014). Enhanced audiovisual integration with aging in speech perception: A height-ened McGurk effect in older adults. Front. Psychol..

[60] Setti A., Burke K.E., Kenny R., Newell F.N. (2013). Susceptibility to a multisensory speech illusion in older persons is driven by perceptual processes. Front. Psychol..

[61] Thompson L.A., Malloy D. (2004). Attention Resources and Visible Speech Encoding in Older and Younger Adults. Exp. Aging Res..

[62] Massaro D.W. (1998). Perceiving Talking Faces: From Speech Perception to a Behavioral Principle.

[63] Diaz M.T., Yalcinbas E. (2021). The neural bases of multimodal sensory integration in older adults. Int. J. Behav. Dev..

[64] Campbell J., Sharma A. (2020). Frontal Cortical Modulation of Temporal Visual Cross-Modal Re-organization in Adults with Hearing Loss. Brain Sci..

[65] Glick H., Sharma A. (2017). Cross-modal plasticity in developmental and age-related hearing loss: Clinical implications. Hear. Res..

[66] Bavelier D., Hirshorn E.A. (2010). I see where you’re hearing: How cross-modal plasticity may exploit homologous brain structures. Nat. Neurosci..

[67] Stropahl M., Debener S. (2017). Auditory cross-modal reorganization in cochlear implant users indicates audio-visual integration. NeuroImage Clin..

[68] Oosterhuis E.J., Slade K., May P.J.C., Nuttall H.E. (2023). Toward an Understanding of Healthy Cognitive Aging: The Importance of Lifestyle in Cognitive Reserve and the Scaffolding Theory of Aging and Cognition. J. Gerontol. Ser. B.

[69] Puschmann S., Daeglau M., Stropahl M., Mirkovic B., Rosemann S., Thiel C.M., Debener S. (2019). Hearing-impaired listeners show increased audiovisual benefit when listening to speech in noise. NeuroImage.

[70] Rosemann S., Thiel C.M. (2018). Audio-visual speech processing in age-related hearing loss: Stronger integration and increased frontal lobe recruitment. NeuroImage.

[71] Basu Mallick D., FMagnotti J., SBeauchamp M. (2015). Variability and stability in the McGurk effect: Contributions of participants, stimuli, time, and response type. Psychon. Bull. Rev..

[72] Dully J., McGovern D.P., O’connell R.G. (2018). The impact of natural aging on computational and neural indices of perceptual decision making: A review. Behav. Brain Res..

[73] Sommers M.S., Tye-Murray N., Spehar B. (2005). Auditory-Visual Speech Perception and Auditory-Visual Enhancement in Normal-Hearing Younger and Older Adults. Ear Hear..

[74] Van Engen K.J., Dey A., Sommers M.S., Peelle J.E. (2022). Audiovisual speech perception: Moving beyond McGurk. J. Acoust. Soc. Am..

[75] Alsius A., Paré M., Munhall K.G. (2018). Forty Years After Hearing Lips and Seeing Voices: The McGurk Effect Revisited. Multisensory Res..

[76] Getz L.M., Toscano J.C. (2021). Rethinking the McGurk effect as a perceptual illusion. Atten. Percept. Psychophys..

[77] Massaro D.W. The McGurk effect: Auditory visual speech perception’s piltdown man. Proceedings of the 14th International Conference on Auditory-Visual Speech Processing 2017.

[78] Peelle J.E., Spehar B., Jones M.S., McConkey S., Myerson J., Hale S., Sommers M.S., Tye-Murray N. (2022). Increased Connectivity among Sensory and Motor Regions during Visual and Audiovisual Speech Perception. J. Neurosci..

[79] Tye-Murray N., Spehar B., Myerson J., Hale S., Sommers M. (2016). Lipreading and audiovisual speech recognition across the adult lifespan: Implications for audiovisual integration. Psychol. Aging.

[80] Begau A., Klatt L.I., Schneider D., Wascher E., Getzmann S. (2022). The role of informational content of visual speech in an audiovisual cocktail party: Evidence from cortical oscillations in young and old participants. Eur. J. Neurosci..

[81] van Wassenhove V., Grant K.W., Poeppel D. (2005). Visual speech speeds up the neural processing of auditory speech. Proc. Natl. Acad. Sci. USA.

[82] Stevenson R.A., Nelms C.E., Baum S.H., Zurkovsky L., Barense M.D., Newhouse P.A., Wallace M.T. (2015). Deficits in audiovisual speech perception in normal aging emerge at the level of whole-word recognition. Neurobiol. Aging.

[83] Winneke A.H., Phillips N.A. (2011). Does audiovisual speech offer a fountain of youth for old ears? An event-related brain potential study of age differences in audiovisual speech perception. Psychol. Aging.

[84] Begau A., Klatt L.I., Wascher E., Schneider D., Getzmann S. (2021). Do congruent lip movements facilitate speech pro-cessing in a dynamic audiovisual multi-talker scenario? An ERP study with older and younger adults. Behav. Brain Res..

[85] Tye-Murray N., Sommers M., Spehar B., Myerson J., Hale S. (2010). Aging, Audiovisual Integration, and the Principle of Inverse Effectiveness. Ear Hear..

[86] Gordon M.S., Allen S. (2009). Audiovisual Speech in Older and Younger Adults: Integrating a Distorted Visual Signal With Speech in Noise. Exp. Aging Res..

[87] Dey A., Sommers M.S. (2015). Age-related differences in inhibitory control predict audiovisual speech perception. Psychol. Aging.

[88] Sommers M.S., Spehar B., Tye-Murray N., Myerson J., Hale S. (2020). Age Differences in the Effects of Speaking Rate on Auditory, Visual, and Auditory-Visual Speech Perception. Ear Hear..

[89] Mahoney J.R., Cotton K., Verghese J. (2019). Multisensory Integration Predicts Balance and Falls in Older Adults. J. Gerontol. Ser. A.

[90] Hirst R.J., Setti A., De Looze C., Kenny R.A., Newell F.N. (2022). Multisensory integration precision is associated with better cognitive performance over time in older adults: A large-scale exploratory study. Aging Brain.

[91] Mozolic J.L., Hugenschmidt C.E., Peiffer A.M., Laurienti P.J. (2008). Modality-specific selective attention attenuates multisensory integration. Exp. Brain Res..

[92] Posner M.I., Driver J. (1992). The neurobiology of selective attention. Curr. Opin. Neurobiol..

[93] Talsma D., Doty T.J., Woldorff M.G. (2006). Selective Attention and Audiovisual Integration: Is Attending to Both Modalities a Prerequisite for Early Integration?. Cereb. Cortex.

[94] Fairhall S.L., Macaluso E. (2009). Spatial attention can modulate audiovisual integration at multiple cortical and subcortical sites. Eur. J. Neurosci..

[95] Roberts K.L., Allen H.A. (2016). Perception and Cognition in the Ageing Brain: A Brief Review of the Short- and Long-Term Links between Perceptual and Cognitive Decline. Front. Aging Neurosci..

[96] Talsma D. (2015). Predictive coding and multisensory integration: An attentional account of the multisensory mind. Front. Integr. Neurosci..

[97] Campbell J., Nielsen M., LaBrec A., Bean C. (2020). Sensory Inhibition Is Related to Variable Speech Perception in Noise in Adults With Normal Hearing. J. Speech Lang. Hear. Res..

[98] Cherry E.C. (1953). Some experiments on the recognition of speech, with one and with two ears. J. Acoust. Soc. Am..

[99] Schneider B.A., Pichora-Fuller K., Daneman M. (2010). Effects of Senescent Changes in Audition and Cognition on Spoken Language Comprehension. The Aging Auditory System.

[100] Getzmann S., Golob E.J., Wascher E. (2016). Focused and divided attention in a simulated cocktail-party situation: ERP evidence from younger and older adults. Neurobiol. Aging.

[101] Fabiani M., Low K.A., Wee E., Sable J.J., Gratton G. (2006). Reduced Suppression or Labile Memory? Mechanisms of Inefficient Filtering of Irrelevant Information in Older Adults. J. Cogn. Neurosci..

[102] Gazzaley A., Cooney J.W., McEvoy K., Knight R.T., D’Esposito M. (2005). Top-down Enhancement and Suppression of the Magnitude and Speed of Neural Activity. J. Cogn. Neurosci..

[103] Gazzaley A., Clapp W., Kelley J., McEvoy K., Knight R.T., D’Esposito M. (2008). Age-related top-down suppression deficit in the early stages of cortical visual memory processing. Proc. Natl. Acad. Sci. USA.

[104] Stothart G., Kazanina N. (2016). Auditory perception in the aging brain: The role of inhibition and facilitation in early processing. Neurobiol. Aging.

[105] Alain C., Woods D.L. (1999). Age-related changes in processing auditory stimuli during visual attention: Evidence for deficits in inhibitory control and sensory memory. Psychol. Aging.

[106] Wild-Wall N., Falkenstein M. (2010). Age-dependent impairment of auditory processing under spatially focused and divided attention: An electrophysiological study. Biol. Psychol..

[107] Hasher L., Lustig C., Zacks R. (2007). Inhibitory Mechanisms and the Control of Attention. Var. Work. Mem..

[108] Borghini G., Candini M., Filannino C., Hussain M., Walsh V., Romei V., Zokaei N., Cappelletti M. (2018). Alpha Oscillations Are Causally Linked to Inhibitory Abilities in Ageing. J. Neurosci..

[109] Hasher L., Zacks R.T. (1988). Working memory, comprehension, and aging: A review and a new view. Psychol. Learn. Motiv..

[110] Pichora-Fuller M.K., Alain C., Schneider B.A. (2017). Older adults at the cocktail party. The Auditory System at the Cocktail Party.

[111] Getzmann S., Klatt L.I., Schneider D., Begau A., Wascher E. (2020). EEG correlates of spatial shifts of attention in a dynamic multi-talker speech perception scenario in younger and older adults. Hear. Res..

[112] Hugenschmidt C.E., Mozolic J.L., Laurienti P.J. (2009). Suppression of multisensory integration by modality-specific attention in aging. Neuroreport.

[113] Guerreiro M.J.S., Anguera J.A., Mishra J., Van Gerven P.W.M., Gazzaley A. (2014). Age-equivalent Top–Down Modulation during Cross-modal Selective Attention. J. Cogn. Neurosci..

[114] Guerreiro M.J.S., Adam J.J., Van Gerven P.W.M. (2014). Aging and response interference across sensory modalities. Psychon. Bull. Rev..

[115] Guerreiro M.J., Eck J., Moerel M., Evers E.A., Van Gerven P.W. (2015). Top-down modulation of visual and auditory cortical processing in aging. Behav. Brain Res..

[116] Lim S.K., Kong S. (2022). Prevalence, physical characteristics, and fall risk in older adults with and without possible sarcopenia. Aging Clin. Exp. Res..

[117] Callis N. (2016). Falls prevention: Identification of predictive fall risk factors. Appl. Nurs. Res..

[118] Reed-Jones R.J., Solis G.R., Lawson K.A., Loya A.M., Cude-Islas D., Berger C.S. (2013). Vision and falls: A multidisciplinary review of the contributions of visual impairment to falls among older adults. Maturitas.

[119] Campos J., Ramkhalawansingh R., Pichora-Fuller M.K. (2018). Hearing, self-motion perception, mobility, and aging. Hear. Res..

[120] Zhang S., Xu W., Zhu Y., Tian E., Kong W. (2020). Impaired Multisensory Integration Predisposes the Elderly People to Fall: A Systematic Review. Front. Neurosci..

[121] Hupfeld K., McGregor H., Hass C., Pasternak O., Seidler R. (2022). Sensory system-specific associations between brain structure and balance. Neurobiol. Aging.

[122] Osoba M.Y., Rao A.K., Agrawal S.K., Lalwani A.K. (2019). Balance and gait in the elderly: A contemporary review. Laryngoscope Investig. Otolaryngol..

[123] Hickok G., Rogalsky C., Matchin W., Basilakos A., Cai J., Pillay S., Ferrill M., Mickelsen S., Anderson S., Love T. (2018). Neural networks supporting audiovisual integration for speech: A large-scale lesion study. Cortex.

[124] Lajoie Y., Teasdale N., Bard C., Fleury M. (1993). Attentional demands for static and dynamic equilibrium. Exp. Brain Res..

[125] Stapleton J., Setti A., Doheny E.P., Kenny R.A., Newell F.N. (2014). A standing posture is associated with increased susceptibility to the sound-induced flash illusion in fall-prone older adults. Exp. Brain Res..

[126] Scheliga S., Kellermann T., Lampert A., Rolke R., Spehr M., Habel U. (2023). Neural correlates of multisensory integration in the human brain: An ALE meta-analysis. Rev. Neurosci..

[127] Gao C., Green J.J., Yang X., Oh S., Kim J., Shinkareva S.V. (2023). Audiovisual integration in the human brain: A coordinate-based meta-analysis. Cereb. Cortex.

[128] Beauchamp M.S. (2005). See me, hear me, touch me: Multisensory integration in lateral occipital-temporal cortex. Curr. Opin. Neurobiol..

[129] Straube B., Wroblewski A., Jansen A., He Y. (2018). The connectivity signature of co-speech gesture integration: The superior temporal sulcus modulates connectivity between areas related to visual gesture and auditory speech processing. NeuroImage.

[130] Rennig J., Beauchamp M.S. (2022). Intelligibility of audiovisual sentences drives multivoxel response patterns in human superior temporal cortex. NeuroImage.

[131] Beauchamp M.S., Lee K.E., Haxby J.V., Martin A. (2002). Parallel Visual Motion Processing Streams for Manipulable Objects and Human Movements. Neuron.

[132] Wright T.M., Pelphrey K.A., Allison T., McKeown M., McCarthy G. (2003). Polysensory Interactions along Lateral Temporal Regions Evoked by Audiovisual Speech. Cereb. Cortex.

[133] Ross L.A., Molholm S., Butler J.S., Del Bene V.A., Foxe J.J. (2022). Neural correlates of multisensory enhancement in audiovisual narrative speech perception: A fMRI investigation. NeuroImage.

[134] Callan D.E., Jones J.A., Munhall K., Callan A.M., Kroos C., Vatikiotis-Bateson E. (2003). Neural processes underlying perceptual enhancement by visual speech gestures. Neuroreport.

[135] Sekiyama K., Kanno I., Miura S., Sugita Y. (2003). Auditory-visual speech perception examined by fMRI and PET. Neurosci. Res..

[136] Amedi A., Von Kriegstein K., van Atteveldt N., Beauchamp M.S., Naumer M.J. (2005). Functional imaging of human crossmodal identification and object recognition. Exp. Brain Res..

[137] Miceli G., Bartolomeo P., Navarro V. (2022). Cross-modal integration and plasticity in the superior temporal cortex. Handb. Clin. Neurol..

[138] Calvert G.A., Campbell R., Brammer M.J. (2000). Evidence from functional magnetic resonance imaging of crossmodal binding in the human heteromodal cortex. Curr. Biol..

[139] Bushara K.O., Grafman J., Hallett M. (2001). Neural Correlates of Auditory–Visual Stimulus Onset Asynchrony Detection. J. Neurosci..

[140] Johnston P.R., Alain C., McIntosh A.R. (2022). Individual Differences in Multisensory Processing Are Related to Broad Differences in the Balance of Local versus Distributed Information. J. Cogn. Neurosci..

[141] Powers A.R., Hevey M.A., Wallace M.T. (2012). Neural Correlates of Multisensory Perceptual Learning. J. Neurosci..

[142] Szycik G.R., Stadler J., Tempelmann C., Münte T.F. (2012). Examining the McGurk illusion using high-field 7 Tesla functional MRI. Front. Hum. Neurosci..

[143] Beauchamp M.S., Nath A.R., Pasalar S. (2010). fMRI-guided transcranial magnetic stimulation reveals that the superior temporal sulcus is a cortical locus of the McGurk effect. J. Neurosci..

[144] Stevenson R.A., Altieri N.A., Kim S., Pisoni D.B., James T.W. (2010). Neural processing of asynchronous audiovisual speech perception. NeuroImage.

[145] Stevenson R.A., VanDerKlok R.M., Pisoni D.B., James T.W. (2011). Discrete neural substrates underlie complementary audiovisual speech integration processes. NeuroImage.

[146] Tóth B., Farkas D., Urbán G., Szalárdy O., Orosz G., Hunyadi L., Hajdu B., Kovács A., Szabó B.T., Shestopalova L.B. (2019). Attention and speech-processing related functional brain networks activated in a multi-speaker environment. PLoS ONE.

[147] Donoghue T., Schaworonkow N., Voytek B. (2022). Methodological considerations for studying neural oscillations. Eur. J. Neurosci..

[148] Wang X.-J., Lee J.J., Schmit B.D., Bellet J., Chen C.-Y., Hafed Z.M., Hoseini M.S., Pobst J., Clawson W., Shew W. (2010). Neurophysiological and Computational Principles of Cortical Rhythms in Cognition. Physiol. Rev..

[149] Keil J., Senkowski D. (2018). Neural Oscillations Orchestrate Multisensory Processing. Neurosci..

[150] Siegel M., Donner T.H., Engel A.K. (2012). Spectral fingerprints of large-scale neuronal interactions. Nat. Rev. Neurosci..

[151] Lange J., Keil J., Schnitzler A., van Dijk H., Weisz N. (2014). The role of alpha oscillations for illusory perception. Behav. Brain Res..

[152] Kelly S.P., Lalor E.C., Reilly R.B., Foxe J.J. (2006). Increases in Alpha Oscillatory Power Reflect an Active Retinotopic Mechanism for Distracter Suppression During Sustained Visuospatial Attention. J. Neurophysiol..

[153] Keller A.S., Payne L., Sekuler R. (2017). Characterizing the roles of alpha and theta oscillations in multisensory attention. Neuropsychologia.

[154] Romei V., Gross J., Thut G. (2010). On the Role of Prestimulus Alpha Rhythms over Occipito-Parietal Areas in Visual Input Regulation: Correlation or Causation?. J. Neurosci..

[155] Keil J., Senkowski D. (2017). Individual Alpha Frequency Relates to the Sound-Induced Flash Illusion. Multisensory Res..

[156] Foxe J.J., Snyder A.C. (2011). The Role of Alpha-Band Brain Oscillations as a Sensory Suppression Mechanism during Selective Attention. Front. Psychol..

[157] Klimesch W., Sauseng P., Hanslmayr S. (2007). EEG alpha oscillations: The inhibition–timing hypothesis. Brain Res. Rev..

[158] Thut G., Nietzel A., Brandt S.A., Pascual-Leone A. (2006). α-Band Electroencephalographic Activity over Occipital Cortex Indexes Visuospatial Attention Bias and Predicts Visual Target Detection. J. Neurosci..

[159] Rihs T.A., Michel C.M., Thut G. (2009). A bias for posterior α-band power suppression versus enhancement during shifting versus maintenance of spatial attention. NeuroImage.

[160] Rihs T.A., Michel C.M., Thut G. (2007). Mechanisms of selective inhibition in visual spatial attention are indexed by α-band EEG synchronization. Eur. J. Neurosci..

[161] Sauseng P., Klimesch W., Stadler W., Schabus M., Doppelmayr M., Hanslmayr S., Gruber W.R., Birbaumer N. (2005). A shift of visual spatial attention is selectively associated with human EEG alpha activity. Eur. J. Neurosci..

[162] Foxe J.J., Simpson G.V., Ahlfors S.P. (1998). Parieto-occipital ∼1 0Hz activity reflects anticipatory state of visual attention mechanisms. Neuroreport.

[163] Worden M.S., Foxe J.J., Wang N., Simpson G.V. (2000). Anticipatory biasing of visuospatial attention indexed by retinotopically specific α-bank electroencephalography increases over occipital cortex. J. Neurosci..

[164] Posner M.I., Snyder C.R., Davidson B.J. (1980). Attention and the detection of signals. J. Exp. Psychol. Gen..

[165] O’Sullivan A.E., Lim C.Y., Lalor E. (2019). Look at me when I’m talking to you: Selective attention at a multisensory cocktail party can be decoded using stimulus reconstruction and alpha power modulations. Eur. J. Neurosci..

[166] Tune S., Wöstmann M., Obleser J. (2018). Probing the limits of alpha power lateralisation as a neural marker of selective attention in middle-aged and older listeners. Eur. J. Neurosci..

[167] Zanto T.P., Gazzaley A. (2014). Attention and Ageing.

[168] Stern Y., Arenaza-Urquijo E.M., Bartrés-Faz D., Belleville S., Cantilon M., Chetelat G. (2020). Reserve, Resilience and Pro-tective Factors PIA Empirical Definitions and Conceptual Frameworks Workgroup. Whitepaper: Defining and investigating cognitive reserve, brain reserve, and brain maintenance. Alzheimer’s Dement..

[169] Jensen O., Mazaheri A. (2010). Shaping Functional Architecture by Oscillatory Alpha Activity: Gating by Inhibition. Front. Hum. Neurosci..

[170] Ruhnau P., Hauswald A., Weisz N. (2014). Investigating ongoing brain oscillations and their influence on conscious perception–network states and the window to consciousness. Front. Psychol..

[171] Cecere R., Rees G., Romei V. (2015). Individual Differences in Alpha Frequency Drive Crossmodal Illusory Perception. Curr. Biol..

[172] Maurer C., Mergner T., Bolha B., Hlavacka F. (2000). Vestibular, visual, and somatosensory contributions to human control of upright stance. Neurosci. Lett..

[173] Jacobs J.V., Horak F.B. (2007). Cortical control of postural responses. J. Neural Transm..

[174] Ozdemir R.A., Contreras-Vidal J.L., Paloski W.H. (2018). Cortical control of upright stance in elderly. Mech. Ageing Dev..

[175] Edwards A.E., Guven O., Furman M.D., Arshad Q., Bronstein A.M. (2018). Electroencephalographic correlates of continuous postural tasks of increasing difficulty. Neuroscience.

[176] Hülsdünker T., Mierau A., Neeb C., Kleinöder H., Strüder H. (2015). Cortical processes associated with continuous balance control as revealed by EEG spectral power. Neurosci. Lett..

[177] Sipp A.R., Gwin J.T., Makeig S., Ferris D.P., Malcolm B.R., Foxe J.J., Butler J.S., Molholm S., De Sanctis P., Peterson S.M. (2013). Loss of balance during balance beam walking elicits a multifocal theta band electrocortical response. J. Neurophysiol..

[178] Ray W.J., Cole H.W. (1985). EEG Alpha Activity Reflects Attentional Demands, and Beta Activity Reflects Emotional and Cognitive Processes. Science.

[179] Scurry A.N., Lovelady Z., Lemus D.M., Jiang F. (2021). Impoverished inhibitory control exacerbates multisensory impairments in older fallers. Front. Aging Neurosci..

